# 4503 Adaptation of Motor Action in Children with Hemiplegic Cerebral Palsy

**DOI:** 10.1017/cts.2020.289

**Published:** 2020-07-29

**Authors:** Katherine Dimitropoulou, Andrew Gordon

**Affiliations:** 1 Columbia University; 2 Movement Sciences Program, Columbia University

## Abstract

OBJECTIVES/GOALS: We study the association of adaptive decision-making, motor planning, and neuromuscular constraints, in children with hemiplegia. We examine how children scale motor decisions to body mechanics and the distance of a target while reaching in sitting/standing, and if they can recalibrate motor decisions to sudden changes in body mechanics. METHODS/STUDY POPULATION: Forty-five 6-12 year-olds with hemiplegia and 45 age/gender matched typically developing controls participate in clinical tests (i.e. balance, visual perceptual skills, etc.) and 3 experiments. Children “reach to tap” toward a target while sitting with both preferred and not preferred arms under three conditions: regular elbow extension siting and standing and elbow extension range reduced by 50% via a splint while sitting. Trials are easy, ambiguous, and difficult. Motor decisions are compared to abilities and motion sensors (IMUs) worn at wrist, arm, sternum and lumbar area, record biomechanical strategies children use under different decisions. Synchronized video analysis presents biomechanical strategies under different decisions. RESULTS/ANTICIPATED RESULTS: Data collection is still underway. A mixed models analysis is used to compare 2 (group: hemiplegic/typically developing) X 2 (arms: healthy/impaired & dominant/non dominant) X 3 (difficulty levels) the children’s decisions. Functional analysis is used to capture biomechanical strategies children use under different decisions and levels of difficulty. Exploration strategies are recorded relative to levels of difficulty. We will also compute correlations between affordance thresholds for all children and measures of sensation, range of motion, cognition and balance (in each posture). Lastly, a secondary analysis will compare behaviors of children with left/right hemisphere lesions, as they differ in spatial abilities. Preliminary results show that children with hemiplegia make errors with both their affected and unaffected side. DISCUSSION/SIGNIFICANCE OF IMPACT: Motor deficits in children with hemiplegia are the primary focus of treatments. Motor learning interventions focus on biomechanical deficits. Results from these studies expand the focus to planning and cognitive control issues underlying motor deficits.

